# Statistical Analysis Aiming at Predicting Respiratory Tract Disease Hospital Admissions from Environmental Variables in the City of São Paulo

**DOI:** 10.1155/2010/209270

**Published:** 2010-07-18

**Authors:** Micheline de Sousa Zanotti Stagliorio Coêlho, Fabio Luiz Teixeira Gonçalves, Maria do Rosário Dias de Oliveira Latorre

**Affiliations:** ^1^Department of Atmospheric Sciences, Institute of Astronomy, Geophysics and Atmospheric Sciences, University of São Paulo, Rua do Matão, Cidade Universitária, 1226 São Paulo, SP, Brazil; ^2^Laboratory of Experimental Air Pollution, Department of Pathology, School of Medicine, University of São Paulo, São Paulo, Brazil; ^3^Department of Epidemiology, School of Public Health, University of São Paulo, Avenue Dr. Arnaldo, 715, 01246-904 São Paulo, SP, Brazil

## Abstract

This study is aimed at creating a stochastic model, named Brazilian Climate and Health Model (BCHM), through Poisson regression, in order to predict the occurrence of hospital respiratory admissions (for children under thirteen years of age) as a function of air pollutants, meteorological variables, and thermal comfort indices (effective temperatures, ET). The data used in this study were obtained from the city of São Paulo, Brazil, between 1997 and 2000. The respiratory tract diseases were divided into three categories: URI (Upper Respiratory tract diseases), LRI (Lower Respiratory tract diseases), and IP (Influenza and Pneumonia). The overall results of URI, LRI, and IP show clear correlation with SO_2_ and CO, PM_10_ and O_3_, and PM_10_, respectively, and the ETw4 (Effective Temperature) for all the three disease groups. It is extremely important to warn the government of the most populated city in Brazil about the outcome of this study, providing it with valuable information in order to help it better manage its resources on behalf of the whole population of the city of Sao Paulo, especially those with low incomes.

## 1. Introduction

The environmental impact on human health has been observed since Hipocrates, in 400 BC, when, in his book *Air, Water and Places*, he emphasized the importance of environmental conditions, such as atmospheric variables, on diseases and general human health. Nowadays, however, there is another influence which has been caused directly by man, which is air pollution.

The Earth's atmosphere has been contaminated by toxic substances emitted by anthropogenic sources such as vehicles, industries, mining and others since the Industrial Revolution. Such contamination is clearly apparent in large urban centres, like México City and the city of São Paulo, among others.

São Paulo is one of the most polluted areas in the world. According to Böhm et al. [1], Saldiva et al. [2], and Braga [3], air pollution constitutes one of the major sources of public health issues, being responsible for a considerable amount of hospital admissions of patients with respiratory, cardiovascular, stillborns, ophthalmic, dermatological, and haematological diseases [2–9]. According to these studies, it is important to note the association of respiratory morbidity with environmental conditions, such as meteorological variables and, mainly, air pollutants.

This study is aimed at quantifying the impact of environmental variables on respiratory morbidity in the City of São Paulo, in order to prevent them. It should also be noted that respiratory diseases are responsible for around 30% of the total morbidity in Brazil, mainly in the southern and southeastern areas, bringing out the importance of such research (http://www.datasus.gov.br).

Many studies have evaluated the impact of air pollutants and atmospheric conditions on human health over the last sixty years, yielding no hospital admission forecasts nevertheless. In this way, the contribution of this research is to predict respiratory hospital admissions, as well as to consider the effects of Thermal Comfort Indices (TCIs) on them, besides the effects of air pollutants and meteorological variables.

## 2. Material and Method

### 2.1. Data Set

This is an ecological study using time series in the MRSP (Metropolitan Region of São Paulo) during a four-year period (1997–2000). The respiratory morbidity of children under thirteen years old was the dependent and discrete variable of the model, and the independent variables were environmental as follows: air pollutants, meteorological variables, and thermal comfort indices all variables are continuous. The months, days and holidays were variables dummies. The covariates patterns were not necessary, because the data by age and sex were selected before of the modeling.

#### 2.1.1. Respiratory Morbidity Data

Daily records of hospital admissions during the researched period, for children under thirteen years of age, were obtained from Brazil's Ministry of Health. These records related to about 80 hospitals, both public and private, spread over the city of São Paulo, which receive support from the public health system (http://www.datasus.gov.br). Thus, our sample is probably representative of the poorest segment of the population, since most in this category lack private medical care insurance. The classification of the diseases under analysis was based on ICD (*International Code Diseases*, 9th and 10th revisions) and was categorized as follows

- (**URI**): upper respiratory Infections (J00–J06), and other diseases of the upper respiratory tract (J30–J39**)**;
- (**LRI**): lower respiratory infections (J20–J22) and Chronic lower respiratory diseases (J40–J47);
- (**IP**): Influenza and Pneumonia (J10–J18).

#### 2.1.2. Air Pollutant Data

Daily records of PM_10_, CO, SO_2_, and O_3_ were obtained from CETESB (Company of Technology of Environmental Sanitation).

#### 2.1.3. Meteorological Data

Daily records of air temperature, wind velocity, relative humidity, atmospheric pressure, and precipitation were obtained from the Meteorological Station at *Parque Estadual das Fontes do Ipiranga (São Paulo).*

#### 2.1.4. Thermal Comfort Indices Data

In order to have a clearer picture of the effect of meteorological variables on respiratory morbidity, thermal comfort indices (TCIs) were computed and added to the meteorological data set, as done by Gonçalves et al. [8]. Cold and heat stress associated with low and high humidity can be strong stressors on heart patients. Thermal comfort indices deal with two or more meteorological variables (temperature, humidity, and wind, for instance), given that the human body reacts to all environmental variables at the same time. Thermal comfort indices become a useful tool to measure thermal stress, mainly in diseased people, hence their use in this research.

Historically, thermal comfort analysis was based on two specific biometeorological indices, according to Fanger [10], and the impact on human physiology was evaluated, as described by Kalkstein and Valimont [11]. In a thermally comfortable environment, no thermal stress should be experienced. This situation is called thermal neutrality, and as such, no action needs to be taken to maintain the proper heat balance of the body, according to Fanger [10], who provides a sufficiently broad discussion of this topic in his book and defines thermal comfort as “that condition of mind which expresses satisfaction with the thermal environment.” In the present study, the main meteorological variables studied were air temperature, relative humidity, and wind velocity. 

In order to determine the thermal classification, different indices were used, based on the work of several authors, as follows. Ono and Kawamura [12] developed the effective temperature concept, based on individual sensitivity/sensitization, combining air temperature and relative humidity. The following equation was used to determine the effective temperature

(a) $$ET=T-0.4\left[\left(1-\frac{RH}{100}\right)\left(T-10\right)\right],$$

where *T* is (mean, maximum, or minimum) air temperature in °C,* RH* is (mean, maximum, or minimum) relative humidity in %, and *ET* is the effective temperature in °C.

Another index, from Suping et al. [13], is defined as follows:

(b) $$\begin{array}{cc} ET_{w} & =37-\frac{\left(37-T\right)}{\left[0.68-0.0014RH+\left(1/1.76+1.4v^{0.75}\right)\right]}\\ & \;-0.29T\left(1-\frac{RH}{100}\right) \end{array},$$

where *v* is the mean wind velocity in m/s, *T* is (mean, maximum, or minimum) air temperature in °C, *RH* is (mean, maximum, or minimum) relative humidity in %, and *ET_w_* is the effective temperature with wind in °C.

These indices were calculated from data collected at the Meteorological Station at Parque Estadual das Fontes do Ipiranga. It was decided that the following combinations of temperature (mean, maximum, and minimum), air relative humidity (mean, maximum, and minimum), and mean wind data would be employed, in order to verify whether there was a correlation between an increase on hospital admissions and thermal stress: hot and dry (*ET1*), hot and wet (*ET2*), mean (*ET3*), cold and wet (*ET4*), and finally cold and dry (*ET5*). Such combination was performed for both indices *ET* and *ET_w_*.

 In the absence of empirical outdoor thermal comfort studies, it has been widely assumed that the indoor thermal comfort theory applies to outdoor settings without modification [14]. Besides, due to the nonexistence of a Brazilian thermal comfort index, the indexes above, which are considered adequate for Brazil, according to Maia and Gonçalves [15], were adopted in this study.

### 2.2. Statistical Tools

This research took advantage of Poisson Regression Multiple Model (PRMM) for daily admission modeling, as it is the statistical approach recommended for this kind of study [2, 3, 9]. Firstly, a lag structure was applied on all the variables (thermal comfort indices, pollutants, and meteorological variables), ranging from one to seven days, at synoptical scale, to verify which lag had more statistically significant correlation with hospital admissions. The lag structure was used because, for example, air temperature can directly affect hospital respiratory admissions on the same day when it is measured and also on several subsequent days. Finally, the Poisson regression modeling was deployed.

The model was estimated and adjusted by its seasonality (nonparametric function), as well as by season of the year, holidays, days of the week, months, and morbidity of nonrespiratory diseases [16]. The Poisson distribution is assumed as

(1) $$\text{Log}\left[E\left(Y_{p,\text{tci},\text{met}}\right)\right]=\alpha +\beta_{1}X_{p}+\beta_{2}X_{\text{tci}}+\beta_{3}X_{\text{met}}+\varepsilon_{p,\text{tci},\text{met}},$$

where *p*, tci, and met are the air pollutants, thermal comfort, indices and meteorological variables, respectively. *α* and *β* are the equation's coefficients. *X*_*p*_ is the pollutant variable, *X*_tci_ is the thermal comfort index variable, and *X*_met_ is the meteorological variable. *ε* is the error.

Relative Risk (RR) and Admission Increase (AI) with Confidence Interval (CI) were also estimated as follows:

(2) $$\text{RR}=\text{ex}{\text{p}}^{\left(\beta *X\right)},$$

where *X* is the threshold to estimate the independent variable and *β* is the Poisson regression parameter:

(3) $$\text{AI}\;\left(\%\right)=\left[\left(\text{ex}{\text{p}}^{\left(\beta *X\right)}\right)-1\right]*100,$$

(4) $$\text{C}{}_{\text{I}95\%}=\exp\;\;\left[\beta \pm 1.96*\text{se}\left(\beta \right)\right],$$

where se is the standard error for *β*.

To check the fit of the model deviance statistic and the Pearson statistic were used, respectively

(5) $$\begin{array}{c} \text{Dev}=2\overset{K}{\underset{i=1}{\sum }}\left\{y_{i}\ln\;\;\left(\frac{y_{i}}{{\hat{y}}_{i}}\right)+\left(n_{i}-y_{i}\right)\ln\;\;\left(\frac{n_{i}-y_{i}}{n_{i}-{\hat{y}}_{i}}\right)\right\},\\ R_{p}=\frac{\left(y_{i}-i\right)}{\sqrt{i\left(1-i\right)}} \end{array}$$

where *y*_*i*_ value of the discrete variable and *i* is estimative of the model.

The Mean Standard Error (MSE) was used after equations were linearized to check predicting hospital admissions:

(6) $$\text{MSE}=\sqrt{\frac{1}{n}}\overset{n}{\underset{i=1}{\sum }}{\left(P_{i}-O_{i}\right)}^{2},$$

*where* *P*_*i*_ is the admissions estimate, *O*_*i*_ is the admissions that actually occurred and *n* is the the sample.

All analyses were carried on using SPSS 10.0 at a 5% significance level.

## 3. Results and Discussion

### 3.1. Correlation Matrix

Table 1 presents the Pearson correlation matrix for URI, LRI, and IP and meteorological, air pollutant, and thermal comfort indices variables. The proportions of such hospital admissions compared to total hospital admissions been 7.6% (1.1% to LRI, 10% to URI, and 5.9% to IP, resp.).

The variables chosen, in bold, were those with higher statistical significance and correlation coefficients. Based on those results, PRMM was deployed, as shown in Table 2, as follows in Section 3.2. 

To build the models URI, LRI, and IP (Section 3.2), air temperature and relative humidity should not be used separately, as their Pearson correlation coefficient (ETw) is higher than the correlation coefficients obtained by these meteorological variables separately.

### 3.2. Poisson Regression Analysis

Table 2 shows the Poisson coefficients for the three groups: LRI, URI, and IP. In this model, and also according to Mazumdar et al. [16], the control variables were months, days of the week, seasons of the year, and holidays, as usual. In this table, the chosen variables, which presented the highest correlation and statistical significance, were based on a correlation matrix (see Table 1). 

Air pollutants PM_10_ and SO_2_ are presented with similar weights in the PRMM. Therefore, due to the colinearity between both variables, the significance of one decreases the significance of the other, when analysed at the same time. Thus, the selection criterion was to choose those variables with the highest correlation coefficient. 

The pressure and precipitation variables do not present a statistically significant correlation with URI, LRI, and IP.

**Figure 1:**
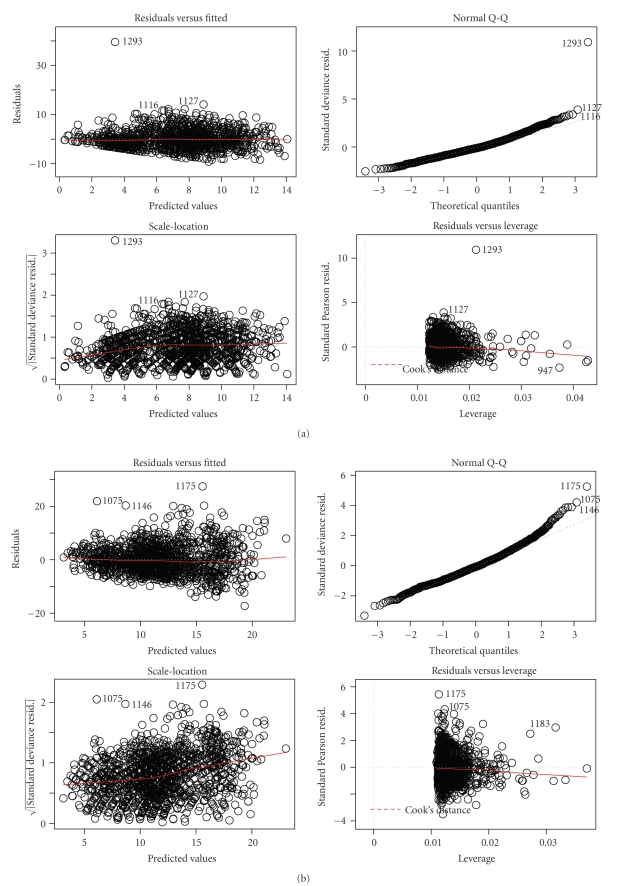

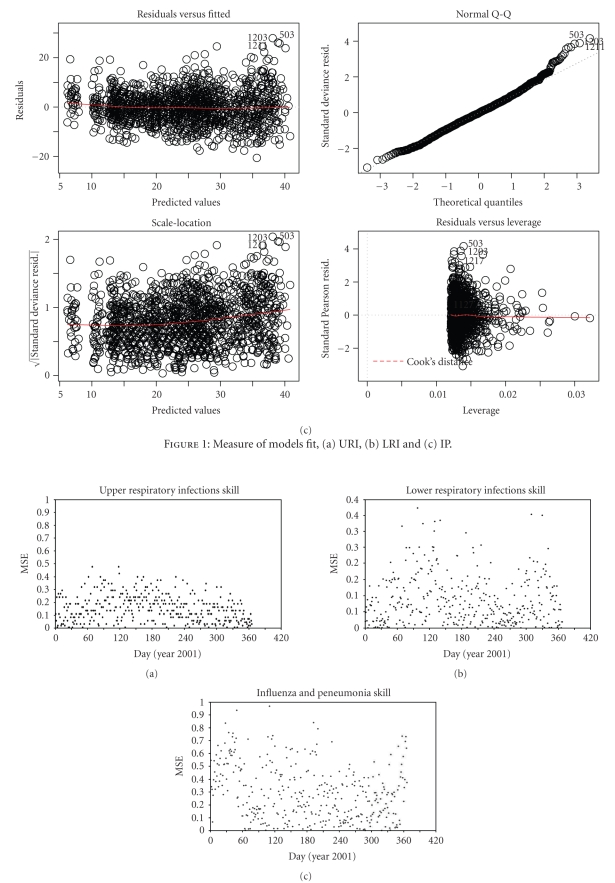
Measure of models fit, (a) URI, (b) LRI and (c) IP.

From (5) (Section 2.2), was obtained for all the three disease categories, in order to evaluate the model's fit through of the pearson statistic (Figure 1).

**Figure 2:**
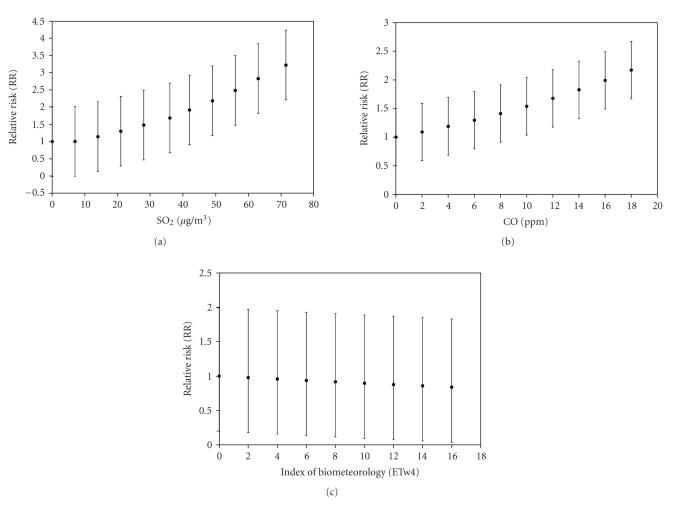
PRMM Skill for (a) URI, (b) LRI and (c) IP.

From (6) (Section 2.2), the Mean Standard Error (MSE) during the year of 2001 was obtained for all the three disease categories, in order to evaluate the model's skill (Figure 2). 

For **URI** morbidity, the most significant variables were SO_2_ and CO with no time lag and ETw4 with a four days time lag (lag4). The seasonal variability of URI is the least among all the disease groups, presenting similar numbers throughout the years, with a slight increase in fall/autumn and winter. During this analysis, SO_2_, even presenting values far below the CETESB safe standard (80 *μ*g/m^3^), is related to an increase in URI morbidity (see Table 1). 

CO also presents a significant correlation coefficient to URI (0.274 at Table 1), despite CETESB's control and the constant decrease in concentration over the last years.

With respect to TCI, *ETw4* presents the highest negative correlation (smaller TCI values, higher respiratory morbidity and vice versa, as expected) with URI compared to the other indices (−0.136) of the same disease group, presenting a lag of four days (lag4). This result means that an arriving cold mass affects children after four days later, which is in accordance with the hospital observations. PRMM for URI presents robust results with mean error around 15%.

For **LRI** morbidity, the most significant variables were PM_10_ with no time lag and O_3_ and ETw4 both with a lag of three days (lag3). This disease group presents a clear seasonality, with higher numbers in winter and fall/autumn. Other seasons also present morbidity although less so.

 In this analysis, PM_10_ and SO_2_ were the most significant air pollutants (0.175 and 0.154, resp.). At PRMM, both pollutants could not be together, because SO_2_ lost its significance while PM_10_ kept it, due to their colinearity. Besides, PM_10_ and O_3_ also presents a significant correlation coefficient (0.093) and might be a LRI predictor. Similar results were found in the MRSP by such other authors as Braga et al. 2002, [17], and Gonçalves et al. 2007. Ozone in the MRSP is generated by vehicular emissions and the presence of VOCs (volatile organic compounds) (Andrade. 1993, [18]). 

In the MRSP, ozone presents a different behavior, that is, higher in springtime, compared to the other pollutants, which are higher in fall/autumn and winter. Frequently, ozone overtakes the CETESB safe standard values (160 *μ*g/m^3^), reaching concentrations as high as 200 *μ*g/m^3^, which characterizes the air as being of very bad quality. Ozone is the only air pollutant which CETESB cannot control properly.

With respect to TCI, again, *ETw4* presents the highest negative correlation with LRI compared to the other indices (−0.187) with a time lag of three days (lag3). It means that ETw4 affects LRI morbidity more than it affects URI morbidity.

PRMM for LRI presents excellent results, as well, with a mean error of less than 30%. Therefore, the LRI model may forecast morbidity for the MRSP in the same way as explained for URI.

With respect to **IP**, the most significant air pollutants were PM_10_ and SO_2_ (0.321 and 0.354, resp.). However, SO_2_ lost significance, and PM_10_ kept it.

With respect to TCI, again, *ETw4* presents a quite strong and the highest negative correlation with IP, compared to the other indices (−0.496), with a time lag of three days (lag3). 

The PRMM deployment for IP presents a mean error of 40%, the worst value among the disease groups, which suggests a new modeling approach. Anyway, estimations can be made, despite the model's limitations. 

### 3.3. Relative Risk and Admission Increase

#### 3.3.1. URI

The model behaves satisfactorily, based on the URI morbidity estimation from the PRMM equation. From these results, it is possible to verify that the increase in respiratory morbidity is due to an increase in SO_2_ or CO or a decrease in ETw4 (see Table 1). From the coefficients found in the PRMM model, it was possible to calculate the increase in URI morbidity, so that an increase of 10 *μ*g/m^3^ in the concentration of SO_2_ is related to a nonlinear behavior in the URI morbidity increase, ranging from 13.9% at low values of SO_2_ to 182.9% at high values of SO_2_.CO also does not show linear behavior, considering that an increase of 2 ppm is related to an increase of 9% at low values of CO to 99% at high values of CO. On the other hand, *ETw4 *presents a linear behaviour, as for every increase of 2°C in TCI, URI morbidity decreases approximately 2.0% (see Table 3). Therefore, colder temperatures imply an increase in URI morbidity. 

**Figure 3:**
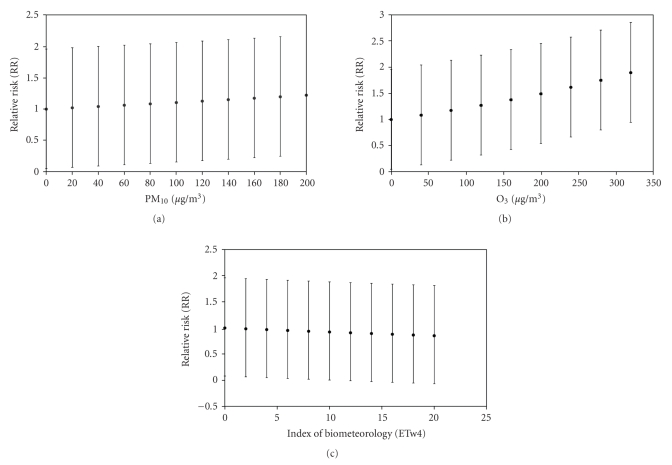
URI Relative Risk (RR) for different explained variables: (a) SO_2_, (b) CO and (c) ETw4.

The Relative Risk rises from 1 to 2.8 with CI_95%_ ranging from −1 to +1 when SO_2_ varies from 0 to 80 *μ*g/m^3^ (see Figure 3(a)). Regarding CO, the Relative Risk rises from 1 to 1.9 with CI_95%_ ranging from −0.6 to +1.0 when CO varies from 0 to 16 ppm (see Figure 3(b)). When it comes to ETw4, the Relative Risk decreases from 1 to 0.8 with CI_95%_ raging from −1.0 to +0.8 when ETw4 varies from 0 to 16°C (see Figure 3(c)).

#### 3.3.2. LRI

The model presents robust results which forecast morbidity with an MSE below 25%. In this case, the variables used in this analysis were PM_10_, O_3_, and ETw4, as shown in Table 4. With respect to PM_10_, an increase of 20 *μ*g/m^3^ indicates a quasilinear increment of LRI, with an average increase of approximately 2.2% in the morbidity of this disease group. Several other authors, such as Zanobetti et al. [19], present similar results. Regarding ozone, which is the air pollutant that has more frequently overpassed safe thresholds in the MRSP [20], an increase of 40 *μ*g/m^3^ did not represent a linear increase in LRI morbidity. With respect to ETw4, an increase of 2°C is related to a linear decrease of approximately 1.5% in the LRI morbidity, bringing out the protection effect of a more comfortable weather (warmer and drier). 

**Figure 4:**
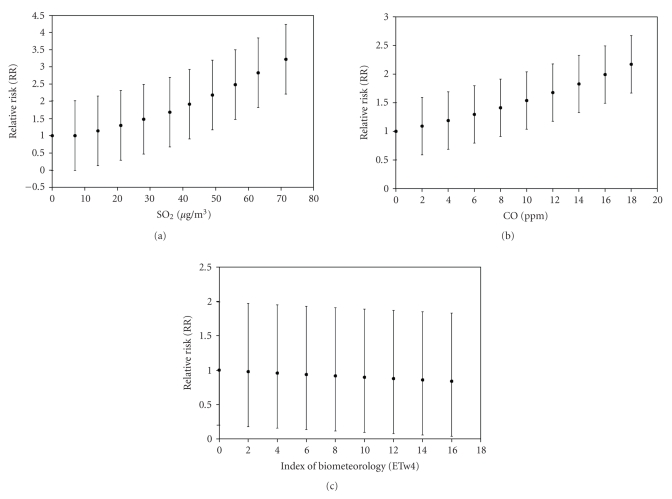
LRI Relative Risk (RR) for different explained variables: (a) PM_10_, (b) ozone and (c) ETw4.

The Relative Risk rises from 1 to 1.2 with CI_95%_ ranging from −1 to +1 when PM_10_ varies from 0 to 160 *μ*g/m^3^ (see Figure 4(a)). Regarding O_3_, the Relative Risk rises from 1 to 2 with CI_95%_ ranging from −1 to +1.0 when O_3_ varies from 0 to 320 *μ*g/m^3^ (see Figure 4(b)). When it comes to ETw4, the Relative Risk decreases from 1 to 0.8 with CI_95%_ ranging from 1.0 to −0.8 when ETw4 varies from 0 to 16°C (see Figure 4(c)).

#### 3.3.3. IP

With regard to IP, the results are similar to the other disease groups so that there was a decrease of IP morbidity in response to an increase in ETw4, as expected. A decrease of 2°C indicates an average increase of 3.3% in IP morbidity, which is considerably higher than the decrease observed in URI and LRI (see Table 4).

On the other hand, an increase in PM_10_ is related to an increase in IP morbidity (see Table 5). A 20 *μ*g/m^3^ increment is related to an average increase of approximately 5% in IP morbidity, which is over twice as high as that in LRI (see Table 4).

**Figure 5:**
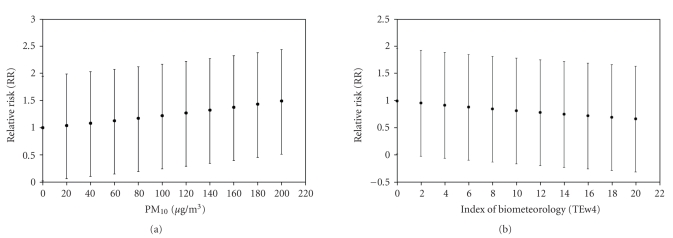
IP Relative Risk (RR) for different explained variables: (a) PM_10_ and (b) ETw4.

The Relative Risk rises from 1 to 1.5 with CI_95%_ ranging from −1 to +1 when PM_10_ varies from 0 to 160 *μ*g/m^3^ (see Figure 5(a)). Regarding ETw4, the Relative Risk decreases from 1 to 0.6 with CI_95%_ ranging from −1.0 to +0.8 when ETw4 varies from 0 to 16°C (see Figure 5(b)).

#### 3.3.4. URI, LRI, and IP Summary

In summary, with respect to air pollutants, URI shows the impact of SO_2_ and CO. With respect to LRI, it is affected by PM_10_ and ozone. With regard to IP, it is affected by PM_10_. Therefore, different air pollutants differently affect each disease group, as expected. With respect to TCI (an index obtained from meteorological variables), ETw4 shows an impact on the three disease groups, more considerably on IP. Also not surprisingly, there is more discomfort from cold and wet weather, which generates higher rates of morbidity.

From the coefficients estimated in the URI, LRI, and IP models, it became possible to predict hospital admission variations as well as their relative risks, according to the individual variation of each associated variable.

The increase in SO_2_ around 80 *μ*m/m^3^, which is a feasible variation in São Paulo, rises in URI hospital admissions and may reach 182.9%. This result is worrying as, according to CETESB, 365 *μ*g/m^3^ is the maximum acceptable level of such air pollutant, which is considerably high. With regard to CO, in case there is a 16 ppm increase, hospital admissions are expected to rise by 99.0%.

With regard to LRI, it was noticed that with an increase in PM_10_ of up to 160 *μ*g/m^3^, which is considered acceptable, admissions will rise by 17.4%, and the Relative Risk will rise from 1 to 1.2. About O_3_, it was noted that if it increases up to 160 *μ*g/m^3^, which is considered acceptable according to CETESB. However the admissions will rise by 37.7%, and the Relative Risk will rise from 1.0 to 2.0. This pollutant has frequently overtaken safe levels of air quality, reaching peaks of 283.4 *μ*m/m^3^, resulting in a hospital admission rise of 75.0%.

Regarding IP, it was observed that if the TCI variation is positive, it behaves as a protector factor, as the higher the index the more comfortable (less cold) is the sensation. Nevertheless, it should be considered that such index tends to be uncomfortable, naturally, as it uses minimum temperatures in its calculation. If ETw4 varies from 0°C to 20.0°C, admissions due to this variation will decrease by 16.1%, 14.8%, and 33% for URI, LRI, and IP, respectively.

## 4. Conclusions

After deploying the PRMM, a hybrid model was built, named Brazilian Climate and Health Model (BCHM), capable of predicting hospital admissions from environmental variables, whose values may either be measured or obtained from a meteorological forecasting mathematical model. Such approach of allowing the use of variables came from a meteorological forecasting mathematical model is similar to the approach adopted by the *Model Output Statistics *(Karl, 1979, [21]).

According to the analysis in this article, it is possible to state that air pollutants, thermal comfort indexes, and meteorological variables show a statistically significant correlation with LRI, URI, and IP hospital admissions.

The projected model for LRI and URI shows robust overall results with an MSE below 25%, while the IP model accuracy was not as good as the other two, with a MSE of approximately 40%. With an error smaller than 30%, it is possible to forecast URI and LRI morbidity based on the thermal comfort indexes and air pollutants measures, as well as based on meteorological variables forecasting. 

Relative Risk results show URI associated with CO and SO_2_, LRI with PM_10_ and Ozone, and IP with PM_10_, as well. ETw4 was also associated with all the disease groups.

On the whole, the models were satisfactory because the objective of this paper was to show that environmental variables could be used to estimate hospital admissions. However, health depends not only on environmental factors, but also on several other factors: hereditary, nutritional, and economic, for instance. This explains part of the model's error. Nevertheless, the BCHM, besides estimating hospital admission from real data, can also be used to predict scenarios resulting from the climate change and extreme weather events (cold and hot air masses). 

## Figures and Tables

**Table 1:** Correlation between air pollutants (PM_10_, SO_2_, CO, NO_2_, and O_3_), meteorological variables (air temperature, relative humidity, pressure, and precipitation), and comfort indices (ET and ETw) and URI, LRI, and IP. The values in bold have higher statistical significance and correlation coefficients.

Independent variables	lag	URI r(p)	LAG	LRI R(p)	LAG	IP R(p)
PM_10_	lag0	0.204 (*P* < .001)	lag0	**0.175** (*P* < .001)	lag0	**0.321** (*P* < .001)
SO_2_	lag0	** 0.313** (*P* < .001)	lag0	0.154 (*P* < .001)	lag0	0.354 (*P* < .001)
CO	lag0	**0.274** (*P* < .001)	lag5	−0.114 (*P* = .001)	lag0	0.181 (*P* < .001)
NO_2_	lag0	0.266 (*P* < .001)	lag0	0.025 (*P* > .005)	lag0	0.188 (*P* < .001)
O_3_	lag0	−0.105 (*P* < .001)	lag3	**0.093** (*P* = .001)	lag7	−0.097 (*P* < .003)
ET1	lag4	−0.129 (*P* < .001)	lag5	−0.133 (*P* = .001)	lag3	−0.267 (*P* < .001)
ET2	lag4	−0.129 (*P* < .001)	lag5	−0.131 (*P* = .001)	lag3	−0.263 (*P* < .001)
ET3	lag4	−0.131 (*P* < .001)	lag4	−0.185 (*P* < .001)	lag3	−0.392 (*P* < .001)
ET4	lag6	−0.134 (*P* < .001)	lag3	−0.186 (*P* < .001)	lag2	−0.445 (*P* < .001)
ET5	lag6	−0.128 (*P* < .001)	lag3	−0.185 (*P* < .001)	lag2	−0.435 (*P* < .001)
ETw1	lag4	−0.135 (*P* < .001)	lag5	−0.135 (*P* = .001)	lag3	−0.287 (*P* < .001)
ETw2	lag4	−0.129 (*P* < .001)	lag5	−0.118 (*P* = .001)	lag3	−0.248 (*P* < .001)
ETw3	lag4	−0.134 (*P* < .001)	lag3	−0.182 (*P* < .001)	lag3	−0.396 (*P* < .001)
ETw4	lag4	−**0.136** (*P* < .001)	lag3	−**0.187** (*P* < .001)	lag3	−**0.496** (*P* < .001)
ETw5	lag4	−0.134 (*P* < .001)	lag3	−0.182 (*P* < .001)	lag3	−0.401 (*P* < .001)
Tmean	lag3	−0.132 (*P* < .001)	lag5	−0.185 (*P* < .001)	lag3	−0.391 (*P* < .001)
Tminimum	lag3	−0.134 (*P* < .001)	lag3	−0.186 (*P* < .001)	lag3	−0.445 (*P* < .001)
Tmaximum	lag4	−0.129 (*P* < .001)	lag5	−0.131 (*P* = .006)	lag2	−0.262 (*P* < .001)
Pressure mean	lag3	0.087 (*P* < .028)	lag2	0.203 (*P* < .001)	lag3	0.378 (*P* < .001)
Pressure minimum	lag3	0.089 (*P* < .032)	lag3	0.200 (*P* < .001)	lag3	0.375 (*P* < .001)
Pressure maximum	lag3	0.084 (*P* < .033)	lag2	0.207 (*P* < .001)	lag2	0.385 (*P* < .001)
RHmean	lag3	0.051 (*P* < .042)	lag1	−0.115 (*P* = .001)	lag0	−0.155 (*P* < .001)
RHminimum	lag5	0.036 (*P* < .065)	lag1	−0.145 (*P* < .001)	lag0	−0.193 (*P* < .001)
RHmaximum	lag4	−0.024 (*P* < .074)	lag1	−0.099 (*P* = .001)	lag4	−0.122 (*P* < .001)
Precipitation	lag3	−0.057 (*P* < .038)	lag3	−0.141 (*P* < .001)	lag0	−0.212 (*P* < .001)

**Table 2:** Poisson Regression model and coefficients for LRI, URI, and IP.

Model		*β*_0_	*β*_1_	*β*_2_	*β*_3_
URI*	poisson	0.916	0.009SO_2_lag0	0.023COlag0	−0.007ETw4lag4
LRI*	poisson	1.661	0.001PM_10_lag0	0.002O_3_lag3	−0.012ETw4lag3
IP*	poisson	3.828	0.002PM_10_lag0	−0.001ETw4lag3	**–—**

*Adjusted for all statistically significant pollutants individual analysis and also adjusted for, days of the week, months, and holidays.

**Table 3:** URI Morbidity increase according to each independent variable.

Variation	Δ1	Δ2	Δ3	Δ4	Δ5	Δ6	Δ7	Δ8
SO_2_ (*μ*g/m^3^)	0–10	0–20	0–30	0–40	0–50	0–60	0–70	0–80
Increase (%)	13.9	29.7	47.7	68.2	91.6	118.1	148.4	182.9
CO (ppm)	0–2	0–4	0–6	0–8	0–10	0–12	0–14	0–16
Increase (%)	9.0	18.8	29.4	41.1	53.7	67.5	82.6	99.0
ETw4 (°C)	0–2	0–4	0–6	0–8	0–10	0–12	0–14	0–16
Decreases (%)	−2.2	−4.3	−6.4	−8.4	−10.4	−12.4	−14.3	−16.1

**Table 4:** LRI Morbidity increase according to each independent variable.

Variation	Δ1	Δ2	Δ3	Δ4	Δ5	Δ6	Δ7	Δ8
PM_10_ (*μ*g/m^3^)	0–20	0–40	0–60	0–80	0–100	0–120	0–140	0–160
Increase (%)	2.0	4.1	6.2	8.3	10.5	12.7	15.0	17.4
Ozone (*μ*g/m^3^)	0–40	0–80	0–120	0–160	0–200	0–240	0–280	0–320
Increase (%)	8.3	17.4	27.1	37.7	49.2	61.6	75.1	89.6
ETw4 (°C)	0–2	0–4	0–6	0–8	0–10	0–12	0–14	0–16
Increase (%)	−1.6	−3.1	−4.7	−6.2	−7.7	−9.2	−10.6	−12.0

**Table 5:** IP Morbidity increase according to each independent variable.

Variation	Δ1	Δ2	Δ3	Δ4	Δ5	Δ6	Δ7	Δ8
PM_10_ (*μ*g/m^3^)	0–20	0–40	0–60	0–80	0–100	0–120	0–140	0–160
Increase (%)	4.1	8.3	12.7	17.4	22.1	27.1	32.3	37.7
ETw4 (°C)	0–2	0–4	0–6	0–8	0–10	0–12	0–14	0–16
Increase (%)	−3.9	−7.7	−11.3	−14.8	−18.1	−21.3	−24.4	−27.4
